# Alopecia areata: descriptive analysis in a Brazilian sample^☆^

**DOI:** 10.1016/j.abd.2021.04.016

**Published:** 2022-07-22

**Authors:** Andressa Sato de Aquino Lopes, Leopoldo Duailibe Nogueira Santos, Mariana de Campos Razé, Rosana Lazzarini

**Affiliations:** Dermatology Clinic, Santa Casa de Misericórdia de São Paulo, São Paulo, SP, Brazil

Dear Editor,

Alopecia areata (AA) is an autoimmune disease, in which CD8+ T-lymphocytes attack the hair follicle in the anagen phase of the hair cycle, causing alopecia.^1^

Clinically, multiple or single, oval, or rounded areas of alopecia are observed. The clinical forms described include plaque, ophiasic, *sisaipho*, diffuse, total, and universal alopecia.^1^

The incidence of this disease in the United States is approximately 2.1%, with a prevalence in the general population of 0.1% to 0.2%.^1^ In Brazil, a study showed that alopecia areata corresponds to 1.2% of dermatological consultations, being the third most diagnosed form of alopecia after androgenetic alopecia and telogen effluvium.^2^ These individuals are more likely to have autoimmune diseases, such as thyroiditis, psoriasis, lupus erythematosus (LE), inflammatory bowel disease, and atopic dermatitis, among others, in addition to the biopsychosocial impact, especially in extensive cases, when patients tend to isolation.^1^, ^3^

The choice of treatment depends on disease extension and patient age.

There are few epidemiological studies in Brazil, which prompted the assessment of cases followed at the dermatology outpatient clinic in a quaternary hospital between 2000 and 2017.

Data were collected retrospectively and submitted to the statistical program R, version 3.4.2. (R Core Team, 2016), descriptively analyzed and compared using Pearson's chi-square test. The study was approved by the Research Ethics Committee under number 29982620.1.0000.5479.

A total of 466 patients were evaluated, of which 272 (58.4%) were women and 194 men (41.6%), with 189 (40.6%) white and 118 (25.3%) non-white individuals, and 159 cases (34.1%) with no information. Women were the most affected, whereas the literature shows no difference between the sexes. This observation reflects the profile of the outpatient clinic, which attends more female patients, who also seek earlier care for hair-related issues.^1^

The mean age was 33.6 years (3 to 86 years), with a standard deviation of 16.6 years. The age at disease onset ranged from 0 to 82 years, with a mean age of 25.6 years (standard deviation of 16.6 years). These data support the observation of other studies, which showed a higher frequency among young adults.^4^

Regarding the extension, the data are listed in Table 1 and show that the higher number of mild cases was similar to those observed in the literature; however, the severe ones showed a lower frequency as compared to earlier reports. This difference can be explained by the fact that the present study demand was spontaneous, while the database, in some studies, is based on the recruitment of patients from more than one institution.^5^

**Table 1:** Distribution of 466 patients with alopecia areata, according to age at onset and sex *versus* severity (affected area).

Age at onset (in years)	Affected area					
	<50%		>50%		Total	
Up to 9	28	43.8%	36	56.3%	64	100.0%
10‒19	50	63.3%	29	36.7%	79	100.0%
20‒29	71	67.0%	35	33.0%	106	100.0%
30‒39	39	59.1%	27	40.9%	66	100.0%
40‒49	34	59.6%	23	40.4%	57	100.0%
50‒59	11	68.8%	5	31.3%	16	100.0%
60‒82	5	31.3%	11	68.8%	16	100.0%
Total	238	58.9%	166	41.1%	404^a^	100.0%

Sex	<50%		>50%		Total	
Female	157	64,0%	88	36,0%	245	100,0%
Male	84	51,5%	79	48,5%	163	100,0%
Total	241	59,1%	167	40,9%	408^b^	100,0%

a Data on 62 patients related to severity are missing.

b Data on 58 patients related to severity are missing.

The severe forms affected more men than women (p < 0.05), similar to a 2017 study. This fact remains to be validated, as there are reports of greater severity among women.^6^

Patients whose dermatosis started before 10 years of age or above 60 years of age had more severe presentations than those with disease onset in other age groups (p < 0.05), as shown in Table 1. The early onset of AA related to greater severity is in agreement with the literature; however, in the group assessed in the present study, patients with late onset disease also had a more severe disease, a fact not described in the literature.^7^, ^8^

The clinical forms of AA observed among the assessed cases are shown in Table 2, and some cases located exclusively on the face were listed in the same table.

**Table 2:** Distribution of 466 patients with AA, according to the clinical forms.

Type of Alopecia areata	Number	%
Plaque	291	62.3
Universal	102	21.9
Ophiasic	28	6.0
Total	16	3.4
Diffuse	5	1.1
*Sisaipho*	1	0.2
**Face**		
Beard	11	2.4
Eyebrows	8	1.7
Eyelashes	1	0.2
Eyebrows + eyelashes	1	0.2
Missing information	3	0.6
**Total**	**466**	**100.0**

The clinical classification of AA should be discussed, since, as demonstrated in the present study, intermediate forms with the involvement of other body areas do not fit the already established criteria. This fact discloses the need for clinical-epidemiological studies for a better understanding.

There was association with another disease in 184 cases (39.5%), some with more than one comorbidity, as described in Table 3. Literature data show 45% of patients with some form of atopy and 19% with thyroid disease.^6^ These associations may be related to the sharing of the same genetic *loci*, immune system cell populations, and cytokine profile present in these diseases.^1^ In this sample, it was not possible to demonstrate a higher frequency of autoimmune diseases among patients with severe forms. Down syndrome is considered a risk for the disease in some studies. However, a recent study highlighted this association as a coincidence, justified by the fact that the studied cases are related to the presence of familial cases and other risk factors.^8^, ^9^

**Table 3:** Distribution of diseases associated with AA in 466 patients.

Disease	Number	%
Thyroid disease	65	14.0
Atopy (asthma, rhinitis, dermatitis)	56	12.0
Down syndrome	15	3.2
Arterial hypertension	12	2.6
Anemia	8	1.7
Vitiligo	8	1.7
Psoriasis	5	1.1
Inflammatory bowel disease	5	1.1
Lupus erythematosus	4	0.9
Diabetes mellitus	4	0.9
Rheumatoid arthritis	2	0.4
No associated diseases	282	60.4
**Total**	**466**	**100.0**

Patients with severe forms were compared with each other regarding sex, presence of autoimmune diseases, atopy, Down syndrome, nail lesions, and presence of familial cases. Pearson's chi-square test showed a statistically significant difference for a higher frequency of severe forms among men (p < 0.05) and for the presence of nail lesions (p = 0.001). The other evaluated parameters did not show significant differences.

Nail alterations were present in 44 patients (9.4%), similar to what is found in the literature (9% and 46%) and seem to be more common in individuals with the severe forms, as in the present study, and related to a worse prognosis.^10^

Sixty-three (13.5%) patients had at least one affected relative and 22 did not know (4.7%). A mother or sibling was affected in 12 cases each (2.6%), an aunt in five cases (1.1%), a son in three (0.6%), an uncle or cousin in two cases each (0.4%), and the father in one case (0.2%). More than one affected relative was observed in four cases (0.9%). The literature shows that familial cases range from 4% to 42%, being more common among first-degree relatives.^5^

AA is a complex disease that affects patient quality of life, with multiple factors involved in its pathogenesis, which are poorly understood. The results of the present study are in agreement with most published investigations, demonstrating the characteristics and profile of patients with this disease in a sample of Brazilian individuals. The impact of the disease on the biopsychosocial aspect was not assessed. The type of study and the absence of a control group limits the statistical power of the results; however, it will be relevant to carry out prospective and case-control studies, which will clarify doubts related to this dermatosis.

## Financial support

None declared.

## Authors' contributions

Andressa Sato de Aquino Lopes: Design and planning of the study; drafting and editing of the manuscript; collection, analysis, and interpretation of data; effective participation in research orientation; intellectual participation in the propaedeutic and/or therapeutic conduct of the studied cases; critical review of the literature; critical review of the manuscript; approval of the final version of the manuscript.

Leopoldo Duailibe Nogueira Santos: Effective participation in research orientation; intellectual participation in the propaedeutic and/or therapeutic conduct of the studied cases; critical review of the literature; critical review of the manuscript; approval of the final version of the manuscript.

Mariana de Campos Razé: Effective participation in research orientation; intellectual participation in the propaedeutic and/or therapeutic conduct of the studied cases; critical review of the literature; critical review of the manuscript; approval of the final version of the manuscript.

Rosana Lazzarini: Design and planning of the study; drafting and editing of the manuscript; collection, analysis, and interpretation of data; effective participation in research orientation; intellectual participation in the propaedeutic and/or therapeutic conduct of the studied cases; critical review of the literature; critical review of the manuscript; approval of the final version of the manuscript.

## Conflicts of interest

None declared.
